# Peers versus professional training of basic life support in Syria: a randomized controlled trial

**DOI:** 10.1186/s12909-018-1241-z

**Published:** 2018-06-18

**Authors:** Fatima Abbas, Bisher Sawaf, Ibrahem Hanafi, Mohammad Younis Hajeer, Mhd Ismael Zakaria, Wafaa Abbas, Fadi Alabdeh, Nazir Ibrahim

**Affiliations:** 1 Faculty of Medicine, Damascus University, Fayez Mansour St. Al-Mezzeh, Damascus, Syria; 2 Faculty of Medicine, Syrian Private University, Damascus, Syria; 3 University of Damascus Dental School, Damascus, Syria; 4 Emergency Department, Damascus Hospital, Damascus, Syria; 5 Syrian Private University, Damascus, Syria

**Keywords:** Basic life support, Cardiopulmonary resuscitation, Medical undergraduate students, Medical training, Disaster medicine, Crisis, Randomized controlled trial

## Abstract

**Background:**

Peer training has been identified as a useful tool for delivering undergraduate training in basic life support (BLS) which is fundamental as an initial response in cases of emergency.

This study aimed to (1) Evaluate the efficacy of peer-led model in basic life support training among medical students in their first three years of study, compared to professional-led training and (2) To assess the efficacy of the course program and students’ satisfaction of peer-led training.

**Methods:**

A randomized controlled trial with blinded assessors was conducted on 72 medical students from the pre-clinical years (1st to 3rd years in Syria) at Syrian Private University. Students were randomly assigned to peer-led or to professional-led training group for one-day-course of basic life support skills. Sixty-four students who underwent checklist based assessment using objective structured clinical examination design (OSCE) (practical assessment of BLS skills) and answered BLS knowledge checkpoint-questionnaire were included in the analysis.

**Results:**

There was no statistically significant difference between the two groups in delivering BLS skills to medical students in practical (*P* = 0.850) and BLS knowledge questionnaire outcomes (*P* = 0.900). Both groups showed statistically significant improvement from pre- to post-course assessment with significant statistical difference in both practical skills and theoretical knowledge (*P*-Value < 0.001). Students were satisfied with the peer model of training.

**Conclusion:**

Peer-led training of basic life support for medical students was beneficial and it provided a quality of education which was as effective as training conducted by professionals. This method is applicable and desirable especially in poor-resource countries and in crisis situation.

**Electronic supplementary material:**

The online version of this article (10.1186/s12909-018-1241-z) contains supplementary material, which is available to authorized users.

## Background

Basic life support (BLS) refers to maintaining an airway and supporting breathing and circulation when sudden cardiac arrest (SCA) occurs, using nothing but a protective mask for rescue breaths [1]. The most important items of BLS are called “the chain of survival” which consists of: 1- Early recognition of SCA and calling for help 2- Early Cardiopulmonary Resuscitation (CPR) 3- Early Defibrillation. Some other simple first aid techniques are usually present in BLS courses and guidelines such as the management of choking and the recovery position. Guidelines highlight the importance of improving BLS by new methods of early Automated External Defibrillation (AED) as well as emergency medical dispatcher (EMD) [1, 2] which are hardly achievable in a country with limited resources. However, when AED is not available, high quality CPR always remains the earliest and most important act to improve outcomes when applied by first aider [1–6]. Immediate CPR can double, triple or quadruple the opportunities of survival when presented effectively [7–12] which is highlighted in the European Resuscitation Council (ERC) recommendations in all its guidelines [1, 2, 13].

In Syria, the crisis in its sixth year continues to devastate all sectors of life, involving the healthcare system. The World Health Organization (WHO) Annual Report of 2015 documented the death of 250,000 while 1.2 million people were injured. There are no accurate estimates of people in need for emergency interventions, but more notable and growing needs during the crisis such as patients with all types of emergencies (Cardiac arrests, massive influx of trauma patients and many other cases) evolved the request for immediate and serious efforts and qualified providers of resuscitation interventions to prevent increased threats and deaths by immediate emergency response [14, 15]. However, the ongoing conflict and the current shortage of resources in Syria represent major obstacles in terms of high costs and the lack of professional training opportunities [16–18].

On the other hand, although training in such a situation is important, and medical students as a part of the healthcare system are more likely to face or act in emergency cases, medical students in Syria are not exposed to any clinical training in emergency in the Faculty. Despite the fact that emergency knowledge is provided later in the final two years as a part of the curriculum, it is provided in theoretical methods while practical training on these aspects is only achieved in postgraduate residency programs. Similarly, emergency courses are not included as a part of curriculum in pre-clinical years of study (The first three years at all Syrian medical faculties) pointing to their emergency response skills that are nearly same as the general public.

This deficiency can be addressed partially with simulation training that can improve the students’ skills and maintain high quality resuscitation [19–21].

Peer teaching (PT) is an effective method in medical education. It is well elaborated on various settings [22–25]. Furthermore, PT can be as effective as professional training [26–28]. While the latter focuses on busy professional trainers for teaching BLS skills, PT can boost effective training by creating smaller interactive groups with confidence and motivation amongst peers [22, 25]. It also encourages students to take a vital role in the educational system in addition to the opportunity by which they can develop their competency. Skills of management, communication and teaching are tiers of experience peers achieve in this model of teaching [29–31]. A review of experience in peer training in BLS supported published studies that proved the value of the concept [27].

Applying PT method in disastrous situations is possible with valuable outcomes [32, 33]. In crisis situations like Syria’s, such a method offers great value and to our knowledge it has not been evaluated in conflict situations similar to Syria’s. Integration of PT in the curriculum in medical faculties is proposed assuming that it may fill the emerging need for clinical training and further enrich the practical skills of medical students, while responding to war contingencies. Furthermore, using BLS-skilled medical students as instructors could serve as an alternative to train the public basics of BLS and therefore improve the emergency response during the crisis.

This study aimed to compare peer to professionals’ training model in BLS course provided to medical students in pre-clinical years, and to evaluate the efficacy and feasibility of BLS training to students at the first years of study by measuring students’ improvement from pre to post training.

## Methods

### Study design

We conducted a parallel-group randomized controlled trial with blinded assessors, at Damascus Hospital in April 2016 to investigate the efficacy of peer-led compared with professional-led BLS training among pre-clinical years’ medical students from the Syrian Private University (SPU).

We opened a call for students from the latest three years (4th, 5th, and 6th) to enroll in BLS training and participate in our study as peers. 12 students entered the course and were trained on BLS skills by the same professionals who led the control group. Students were assessed by the professionals and those who were considered unqualified BLS providers were excluded. Four students were randomly selected and were instructed how to deliver BLS skills and transfer them to other students.

Four professionals (2 emergency doctors, cardiologist and anesthesiologist) led training in the control group. They are experienced in emergency training like BLS, advanced life support (ALS) and other courses in Syrian medical emergency institutes that provide training to wide range of people. Their experience ranges from 3 to 20 years. None of them was considered as an assessor or an author.

Two blinded 10-year experienced professionals in emergency training (trainers in the Syrian resuscitation council) were asked to evaluate students’ skills independently A third assessor who is also professional in emergency training was asked to resolve conflict judgments between live and camera assessment when existed.

### Participants, recruitment and allocation

We asked all students in the pre-clinical years in SPU to participate, 179 students fulfilled the inclusion criteria and signed the written consent form. Exclusion was based on; presence of any health problems preventing students from doing physical exercise, any serious acute or chronic illness (infectious, psychological, physical), scheduling conflict between the date of the BLS course and other faculty’s classes or exams, missing the course or the assessment for any reason, refusing to sign the consent and having any prior experience in BLS skills (previously trained on BLS).

**Fig. 1:**
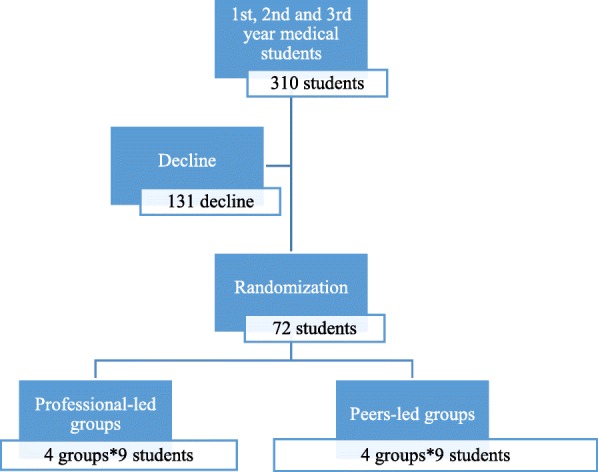
CONSORT diagram, recruitment and allocation

We sampled 72 students and allocated them into the peer and the professional group (36 students in each group). Sample size was chosen in order to conduct similar training to the running emergency courses in Syria and according to the feasibility of conducting the trial within the available resources and the trial circumstances. A multi-stratified disproportioned random sampling of 72 students was done to enroll BLS course by a computer-generated list of random numbers to produce equal distribution across gender and year of study in both groups (Fig.1) (Microsoft Office Excel 2013, Microsoft Corporation, by Impressa Systems, Santa Rosa, California) Randomization and allocation were done by a blinded statistician.

### Course description

Course design was made to be feasible within the deprived resources in the crisis and to be consistent with ERC guidelines [2] with local modifications made by emergency professionals in duration, instructor-to-trainee ratio, course materials, methods to deliver these materials theoretically, and the type of the manikin used to practice CPR. Since the optimal duration for BLS training varies according to the guidelines [34], professionals identified the duration for this course depending on their experience in the Syrian resuscitation council. As to meet the need for more people trained on BLS aspects in Syria during the crisis, number of instructor-to-student in each course is defined as the maximum with respect to the quality of training. One-day-course consisting of 75, 20, 20, 20 min for theoretical BLS, chocking, recovery position, the practical representation of BLS scenario respectively followed by 40-min practical training on BLS skills for each subgroup. Both arms of the study followed the same timeline and no extra time was given to any group. However, both groups had breaks between lectures and students were free to ask and discuss with their instructors.

Learning objectives were explained to all students in the morning at the same time for both groups. Using lectures, videos and simulation scenarios, instructors concentrated on CPR, recovery position and choking. Study materials, all in English, were provided to students during the first lecture at their classes. Same manikins were used for the training and the assessment.

**Fig. 2:**
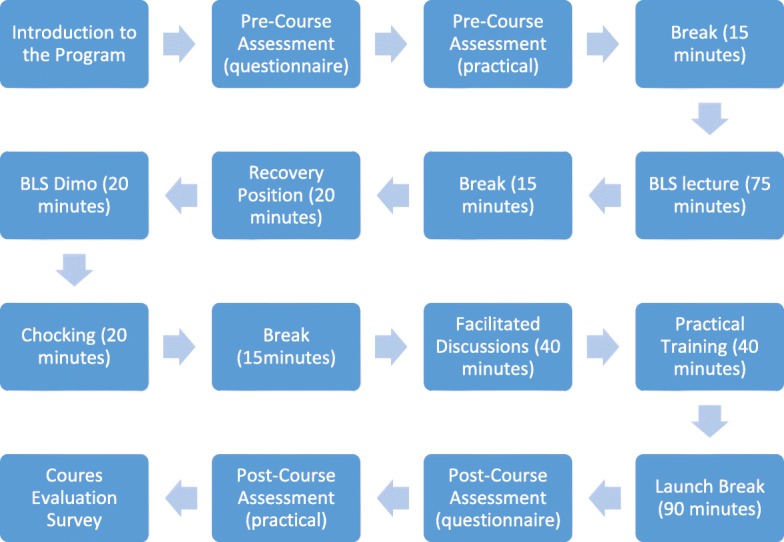
Basic life support course program of the study

Training and assessment were held at the same day for both groups who have been trained on the same aspects and principles and learning objectives. (Fig. 2).

On the day of the experiment students in each arm were divided into 4 subgroups of maximum 9 students, each led by two trainers of BLS skills with a maximum ratio of 2 instructors to 9 students per group (selection of students and instructors for each subgroup was random).

### Assessment

#### BLS practical skills

To ensure that no bias would affect assessment results, we allocated participants randomly to one of two blinded assessors (A or B) to perform a practical simulated scenario assessment using a checklist based evaluation in objective structured clinical examination (OSCE) design. The checklist was constructed in accordance to ERC guideline and it is provided as an additional document (Additional file 1). Students should perform each point correctly to pass the assessment (1- Safe approach, 2- call for help, 3- opening airway, 4-checking cardiopulmonary situation, 5- call ambulance, 6- CPR with effective depth, 7-rate and 8-position, 9-rescue breaths). CPR providers should aim for an inflation duration of about 1 s, with enough volume to make the manikin chest rise, but avoid rapid or forceful breaths. Using the available traditional manikin, effective CPR was considered when chest compressions are performed on the lower half of the sternum, depth should range from 5 to 6 cm causing a voice in the manikin indicating effective compression. Compression to ventilation ratio of 30:2 was considered correct. Trainee should perform BLS skills flowingly.

Each student was assessed twice and independently by the two assessors, directly by assessor A and recorded test was assessed by the assessor B or vice versa.

**Fig. 3:**
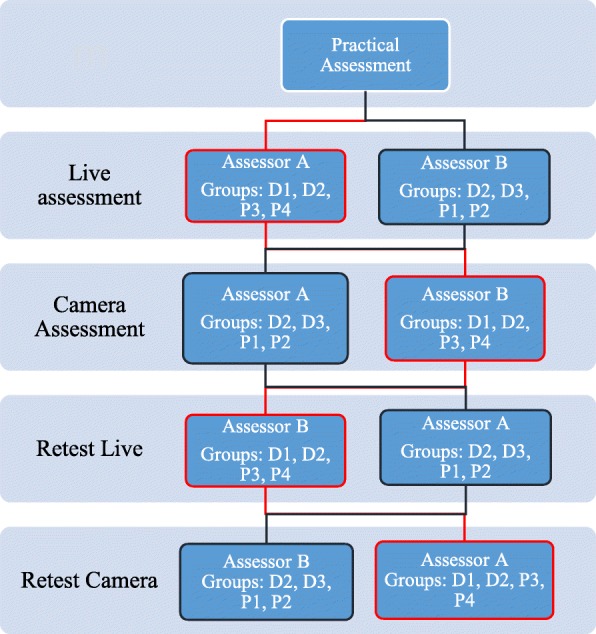
Double check of the practical assessment

Practical test was recorded by 2 cameras; one fixed confronting the manikin and one mobile camera to take the best position confronting the student while doing CPR in the test room as shown in (Fig. 3). Both cameras were checked before the beginning of the course to test the position and accuracy. Video records transferred the whole scenario of each student since his entry to the test room.

### BLS knowledge questionnaire

To assess students’ knowledge we used a 20-item questionnaire with 3 checkpoints per item (60-point-scale) derived from ERC materials after testing the applicability of the questionnaire by a pilot study.

### Students’ evaluation of BLS course survey

We took students’ report using American Heart Association (AHA) survey of BLS course evaluation [35]. Open ended questions were included to investigate their opinions (Table 6).

Both groups underwent pre and a post-course evaluation using the two tools (The checklist and the questionnaire) to assess their improvement in theoretical and practical skills (Fig. 1). In the end of the course, participants were asked for their opinions on peer method, and their satisfaction.

### Outcomes and analysis

Pass percentages were considered as the primary outcome measure to compare the two groups and to evaluate the efficacy of the course. Pass is considered when the trainee completes the practical assessment checklist correctly. Any neglected or wrongly performed step (For example, depth of the compressions or the position) indicates failure. If the trainee failed in the practical test he was given one opportunity to retest and be assessed by the other assessor.

To measure students’ knowledge (theoretical knowledge) 60-point-scale pertained by their answers to the questionnaire was used.

Two-sample T-test was also used to compare questionnaire mean score, pass rates in all stages of the practical assessment between the two groups and to compare between performance of the two genders then one-way ANOVA test was used to analyze effect of year of study on students’ knowledge and attitude toward BLS and lastly Fisher’s exact test was used to analyze how the year of study affects the final results of practical skills using SPSS version 22 (IBM SPSS Statistics for Windows, Version 22.0. Armonk, NY: IBM Corp).

We performed a prospective analysis of pre and post-course outcomes to evaluate the efficacy and feasibility of the BLS course training to pre-clinical medical students. This analysis depended on paired student’s t-test regarding the mean score of questionnaire and the pass rate of the practical assessment using Minitab 16 version (Minitab Inc., State College, Pa, USA).

## Results

Out of the 72 randomized participants, 64 were included in the analysis (30 in the professional-led group and 34 in the peers’ group) excluding 8 students who were absent or late to do the pre-course assessment. All attended the whole course and did all the assessment tools. Characteristics of the participants are summarized in Table 1.

**Table 1:** Basic Sample Characteristics

		Total number	Professional’ Training group	Peers’ Training Group	*P*-value^*;†^
Gender	Males	31	15	16	1.0
	Females	33	15	18	1.0
Study year	1st year	24	12	12	0.872
	2nd year	19	8	11	
	3rd year	21	10	11	

*Significant Level was set at 5%

†Chi square tests were applied

### Comparison of the two groups’ results

There was no significant statistical difference in pass rates (practical performance of BLS skills) between the two groups at any stage of the assessment (live; *p* = 0.333, overall results; *p* = 0.850 and third assessor; *p* = 0.781). The eight failures were distributed equally on the two groups and on the subgroups (Table 2) and a non-significant *p*-value indicated no statistically significant difference in the mean score of the questionnaire (*p* = 0.900) (BLS theoretical knowledge).

**Table 2:** Results of the practical assessment and knowledge questionnaire mean score of trainees between the two groups

		Professionals’ group		Peers’ group		*P*-value*
		Result	*N*	Result	*N*	
Overall pass rates		26	30	30	34	1.000^†^
Live pass rates		27	30	33	34	0.333^†^
Retest overall pass rates		2	6	7	8	0.091^†^
Retest live pass rates		3	6	7	8	0.245^†^
Conflicted pass rates		2	3	9	12	1.000^†^
Questionnaire means	Pre-course	37.6	30	39.26	34	0.080^‡^
	Post-course	53.47	30	53.56	34	0.900^‡^

*Significant Level was set at 5%

†Fisher’s exact test was applied

‡Two-sample T-test was applied

All students were with no experience in BLS skills since all failed the practical test too (0% pass the pre-course assessment) (Table 2).

### The prospective analysis

The BLS course program showed significant improvement regarding both the practical (56 passed the post-course test out of 64 students who attended the course in the both groups, difference = 87%, *p* < 0.001) and applied questionnaire tests prior and after the course with a mean difference of approximately 15 additional gained points in the post course questionnaire score (*p* < 0.001; Table 3).

**Table 3:** Course effectiveness in improving BLS knowledge and practical skills according to Students’ assessments pre and post course (Total Number = 64 in all tests)

	Pre-Course	Post-Course	Difference	*P*-value*
Questionnaire mean score	38.484	53.516	15.031	< 0.001^†^
Practical test pass	0	56		< 0.001^‡^

*Significant Level was set at 5%

†Two-sample T-test was applied

‡Chi square test was applied

**Fig. 4:**
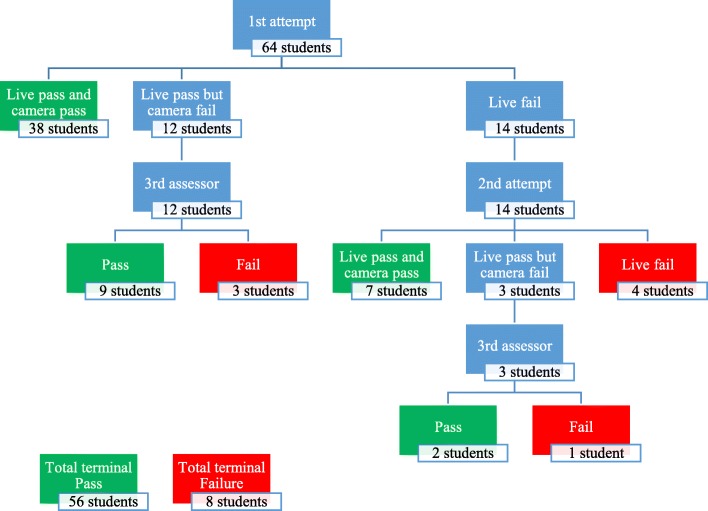
Students’ results of the practical assessment in all its stages

Final results revealed 56 pass in total (85% of all students); 38 passed with no retest or conflict, 7 retested and passed without conflict, 11 passed according to the third assessor’s result as shown in (Table 2 and Fig. 4).

### Affecting factors

There was no statistically significant difference between males and females in the pass rates; in the final results (*p* = 0.925) or live assessment (*p* = 0.275; Table 4). Study year did not affect students’ response to the knowledge questionnaire as no difference between the three years in the mean score shown in Table 5. Study year also seemed not to affect practical test results (Table 5).

**Table 4:** Differences between males and females regarding post course assessment

	Males		Females		*P*-value^*^
	result	number	result	number	
Live overall post-course	28	31	32	33	0.347^†^
Final post-course	27	31	29	33	1.000

*Significant Level was set at 5%

†Fisher’s exact tests were applied

**Table 5:** Participants’ pre- and post-course assessments according to their study year

	First year		Second year		Third year		*P*-value^*^
	Result	Number	Result	Number	Result	Number	
Questionnaire Pre-course	37.75	24	39.316	19	38.571	21	0.414^†^
Questionnaire Post-course	53.125	24	53.632	19	53.857	21	0.680^†^
Overall live Practical Post-course	23	24	18	19	19	21	0.743^‡^
Final practical post-course	20	24	17	19	19	21	0.734^‡^

*Significant Level was set at 5%

*† ANOVA test was applied*

*‡ Pearson Chi-square test was applied*

### AHA survey results (students’ reported evaluation of the course)

Comparing between students’ reported outcomes in both groups shown in (Table 6), reports yielded equivalent results in evaluating the course with no significant differences. However, there was a statistical difference between the two groups in two items (taking the course to obtain professional credit was highly reported in peers’ group and the estimated level of the course; 6 students reported the course to be too easy in the professionals’ group).

**Table 6:** Course evaluation survey response: Positive response for each item

Question	Professionals’ group (total = 30)	Peers’ group (total = 34)	*P*-value*
Provided instruction and help during my skills practice session?	30	34	–
Answered all of my questions before my skills test?	27	32	0.659^†^
Was professional and courteous to the students?	30	34	–
The course learning objectives were clear?	29	32	1.000
The overall level of difficulty of the course was? ^a^	34	24	0.008
The content was presented clearly?	30	34	–
The quality of videos and written materials was? ^b^	25	24	0.383
The equipment was clean and in good working condition?	30	33	1.000
The course prepared me to successfully pass the skills session?	30	34	–
I am confident I can use the skills the course taught me?	30	31	0.241
I will respond in an emergency because of the skills I learned in this course.	28	33	0.559
I took this course to obtain professional education credit or continuing education credit?	12	28	0.001

*Total number = 64*

*Significant Level was set at 5%

†Chi square tests were applied

^a^The positive response (yes) was considered for the choice (appropriate)

^b^The positive response (yes) was considered for the choice (excellent)

Students in the peers group in open-end-questions reported greater motivation and enthusiasm. They were comfortable to discuss and ask their instructors to practice more on BLS skills within the given time. All students in both groups reported confidence when applying BLS skills. Students in the peer group reported satisfaction of PT.

## Discussion

This is the first study of its kind in Syria to compare peer-led with professional-led training in BLS in the settings of crisis. Lack of competent staffs, time to cover more people with training programs, scheduling conflict for clinical instructors between training and patient care, the augmented need for emergency interventions in time of conflicts, beside the cost, all these barriers necessitated an alternative approach to overcome related obstacles.

Evaluation of quality of peer-led training is difficult especially in the poor-resource settings like in Syria. Therefore application of BLS course for medical students in pre-clinical years imposed the importance of using valid and unbiased assessment tools for the two groups. Different methods of assessment were described in published data [36]; we used multiple methods in assessment starting from a practical test with checklist based OSCE, a questionnaire to evaluate knowledge and attitude and lastly a self-reported survey to investigate students’ confidence and satisfaction.

Comparing primary outcomes in the two groups in our study in practical and theoretical aspects (BLS knowledge) confirms that PT is as effective as professional training in crisis situation within the limited resources. Pass rates in the intervention (peers) group were the same as in the professional-trained group with no significant difference. This indicates the ability of trained medical students to transfer high quality skills (correctly performed) to other students effectively. Although pass rates in live assessment were better for peers group initially, number of students who needed retest was higher in peers’ group with no significant difference. However, students who passed the test in both groups performed high quality CPR in depth, rate and position. In the study of Fujiwara et al. [26], students were reported to be potent instructor in BLS training with similar results to what we concluded in our study.

Students’ knowledge in BLS did not differ between the two groups too, neither in pre-course questionnaire response nor in the post-course evaluation when comparing means of questionnaire points. This indicates that the peers were parallel to professional trainers in providing useful information about BLS to the students. Although some research studies suggested that peer-model training is in some occasions better than traditional (professional-led) training [28], in our study the two groups achieved the same results for the primary and secondary outcomes. Using the same materials for both groups possibly contributed in having similar results.

Students in both groups were confident with applying BLS skills. They were similar in their reporting of satisfaction on the material, trainers’ teaching ability, interactive learning via discussions with trainers, presenting the content of the course, and their willing to respond for emergency call. As shown in Table 6. Analyzing the reports of all these variables yielded that PT was satisfactory for students in our study. Students had great motivation and interaction toward learning skills in the peers’ group, and they felt more comfortable to ask, discuss, practice and make mistakes more than those in the professional group.

These findings may have resulted from students’ comfort with peer training. Additionally, the better use of resources by peers such as applying modern presentation techniques and showing videos has positively injected their training with motivation and enjoyment. Nevertheless, professionals were able to answer complex questions about special conditions and cases in emergency with more ease and experience which was sometimes lacking among peer trainers.

The significant improvement from pre to post course manifested among both groups indicates that the course program improved students’ performance in BLS skills pointing to its potency to achieve the targeted outcomes.

Moreover, it proves that medical students in their pre-clinical years are capable of learning effective BLS skills since this training focuses on practical skills rather than scientific basis as many studies yielded [26, 37–39].

Since significant heterogeneity between the two groups was avoided by randomizing students according to their year and gender, there was no difference between the improvements of both groups not in pass rates nor the means of the questionnaire scores (Table 1, Table 2, Table 3 and Table 5). Similar results between the two genders contradict with the suggested evidence that gender may affect BLS skills performance [40]. Enthusiasm and the strict program of the training course along with the randomization are possible reasons to eliminate this effect. There was no effect related to the level of study (year) on BLS skills assuming that BLS courses are applicable to students in medical faculties regardless their previous or progressive knowledge.

### Limitation

Immediate assessment for students after they were trained on BLS didn’t allow investigating the long-term quality of student’s gained skills.

Since physical and scientific ability may have a potential role in making qualified BLS maneuvers; weight, Body mass index (BMI) and annual average of students may have influenced their performance. Although results show no difference between the two groups at any stage of the study concerning year of study and gender, it would have been better to do wider multi-stratified randomization by taking detailed information of students which wasn’t applicable at the time of allocation or the small sample in BLS courses.

In Crisis situation it should have been necessary to evaluate students’ skills in training BLS course to the public. Since medical students in the first years of study have no clinical or emergency skills, they can be considered part of the general population, however, further studies should be conducted in wider spectrum.

From another perspective, we did not find trusted published data regarding the impact of the Syrian crisis on the health care systems or the cases of emergency that need resuscitation skills.

## Conclusion

Our trial showed that peer-led training in BLS for medical students in pre-clinical years is feasible and as effective as health professional-led training. Findings suggest that it can be successfully implemented in countries with limited resources and in situations of crisis such as in Syria. Our findings show that medical students are valuable resources to increase BLS skilled individuals in the community. Integrating BLS training programs to the curriculum will enhance medical training in critical care and may contribute in improvements of the medical emergency responses in Syria.

## Additional files

Additional file 1: BLS checklist for practical test: This checklist contains the essential steps of performing BLS skills according to ERC guidelines. Each student should perform all steps correctly to pass. (PDF 53 kb)

## Abbreviations

AED: Automated External Defibrillation
AHA: American Heart Association
ALS: Advanced life support
BLS: Basic life support
BMI: Body mass index
CPR: Cardiopulmonary Resuscitation
EMD: Emergency medical dispatcher
ERC: European Resuscitation Council
IRB: The institutional review board
OSCE: Objective structured clinical examination
PT: Peer teaching
SCA: Sudden cardiac arrest
SPU: The Syrian Private University
WHO: World Health Organization
