# Supplementary material for: Peers versus professional training of basic life support in Syria: a randomized controlled trial
Source: BMC Med Educ. 2018 Jun 18;18:142. doi: 10.1186/s12909-018-1241-z (PMC6006697; doi:10.1186/s12909-018-1241-z)
Supplement: Supplementary file 1 — BLS checklist for practical test: This checklist contains the essential steps of performing BLS skills according to ERC guidelines. Each student should perform all steps correctly to pass. (PDF 53 kb) [file 12909_2018_1241_MOESM1_ESM.pdf]

BASIC LIFE SUPPORT TEST

Interventions :	Competent	Not competent
**Safe approach .**		
**Stimulate the patient .**		
Shout for help .		
**Open airway .**		
**Look – listen – feel for not more than 10 s .**		
**Call ambulance .**		
**30 chest compressions(depth , position). 100 / M .**		
**2 rescuer breath (not more than 5 s) .**		
**Ratio 30 / 2 :**		
**COMMENTS :**		
**RESULT :**		
**Pass :**	**Retest :**	**Failed :**
